# Report of the Henry Phipps Institute

**Published:** 1907-10

**Authors:** 


### EDITORIAL NOTES AND COMMENTS.

The Business Office of the JOURNAL-RECORD is 1 1-2 to 5 1-2 South Broad Street.

The Editorial Office is 318-319-320 The Prudential.

Address all Business Communications to Dr. George Brown, Manager.

Make remittances payable to THE JOURNAL-RECORD OF MEDICINE.

On matters pertaining to the Editorial and Original communications, address Dr. Bernard Wolff, Atlanta.

Reprints of original articles will be furnished at cost price. Requests for the same should always be made on THE MANUSCRIPT.

We will present, postpaid, on request, to each contributor of an original article, twenty (20) marked copies of THE JOURNAL-RECORD OF MEDICINE containing such article

The September issue of Everybody's Magazine contains some interesting articles upon "Nature Fakers." President Roosevelt reviews the work of some of the notable malefactors and holds up to ridicule many of their absurd statements. The claim has been made that a wolf could reach the heart of a caribou, or a moose, or a horse, at a single bite, and later the same writer describes a double contest between a wolf and a lynx, or a bulldog in which the latter survives twenty slashing bites. Concerning this, Roosevelt says that a wolf that could bite into the heart of a horse would swallow a bulldog or a lynx like a pill.

Edward B. Clarke gives the opinion of many of the leading naturalists in the United States to show that a large number of the statement made by certain nature writers as truth are pure fiction.

### REPORT OF THE HENRY PHIPPS INSTITUTE.

The Third Annual Report of the Henry Phipps Institute for the study, treatment, and prevention of tuberculosis has been received and is not only interesting, but contains a vast amount of valuable scientific knowledge recently acquired by investigators in this important field of research.

The number of patients which forms the basis of this report is smaller than that of the preceding years, but there is a proportionate increase in the scientific work. During the year, twelve hundred and fifty-one patients were admitted for treatment.

A striking feature is the prevalence of tuberculosis in the Hebrew race. The statistics show that alcohol neither causes, prevents or cures tuberculosis. They assert that it looks very much as if the two arch-enemies of the human family, alcoholism and tuberculosis, will have to stand each on its own demerits, and be met by sociologists on independent bases. We will be able to fight these enemies better when we better understand them, and no longer formulate our plans of attack upon untruth.

An interesting occurrence is atrophy of the scapular muscle which in all probability is due, in most cases at least, to an attack of tuberculosis earlier in life from which recovery has in part or in whole taken place. It is observed more frequently upon the right side than upon the left. The beginning of tuberculosis is assigned to the right apex in nearly 66 per cent. of the patients. The report states that it is generally accepted at the present time that the tubercle bacillus gain entrance into the organism through the lymphatic system, whether the port of entry be the respiratory tract, the alimentary canal, the skin or any other part of the body.

About one-half of the patients who applied for treatment had hemoptysis at one time or another.

Focal interstitial nephritis is a condition described as small areas having the appearance of general interstitial nephritis—probably due to healed tubercles.

Foreign-born citizens, it is claimed, bring us nearly one-half the burden of the crusade against tuberculosis, and strange to say foreign-born men give nearly twice the burden of foreign-born women.

